# Metatranscriptomic analyses of honey bee colonies

**DOI:** 10.3389/fgene.2015.00100

**Published:** 2015-03-19

**Authors:** Cansu Ö. Tozkar, Meral Kence, Aykut Kence, Qiang Huang, Jay D. Evans

**Affiliations:** ^1^Ecological Genetics Laboratory, Department of Biological Sciences, Middle East Technical UniversityAnkara, Turkey; ^2^Bee Research Laboratory, United States Department of Agriculture-Agricultural Research ServiceBeltsville, MD, USA

**Keywords:** *Apis mellifera*, pollination, colony collapse disorder, RNA sequencing, bioinformatics, honey bee viruses, trypanosomes

## Abstract

Honey bees face numerous biotic threats from viruses to bacteria, fungi, protists, and mites. Here we describe a thorough analysis of microbes harbored by worker honey bees collected from field colonies in geographically distinct regions of Turkey. Turkey is one of the World's most important centers of apiculture, harboring five subspecies of *Apis mellifera* L., approximately 20% of the honey bee subspecies in the world. We use deep ILLUMINA-based RNA sequencing to capture RNA species for the honey bee and a sampling of all non-endogenous species carried by bees. After trimming and mapping these reads to the honey bee genome, approximately 10% of the sequences (9–10 million reads per library) remained. These were then mapped to a curated set of public sequences containing ca. Sixty megabase-pairs of sequence representing known microbial species associated with honey bees. Levels of key honey bee pathogens were confirmed using quantitative PCR screens. We contrast microbial matches across different sites in Turkey, showing new country recordings of Lake Sinai virus, two *Spiroplasma* bacterium species, symbionts *Candidatus Schmidhempelia bombi*, *Frischella perrara*, *Snodgrassella alvi*, *Gilliamella apicola*, *Lactobacillus* spp.), neogregarines, and a trypanosome species. By using metagenomic analysis, this study also reveals deep molecular evidence for the presence of bacterial pathogens (*Melissococcus plutonius*, *Paenibacillus larvae*), *Varroa destructor*-1 virus, Sacbrood virus, and fungi. Despite this effort we did not detect KBV, SBPV, Tobacco ringspot virus, VdMLV (Varroa Macula like virus), *Acarapis* spp., *Tropilaeleps* spp. and *Apocephalus* (phorid fly). We discuss possible impacts of management practices and honey bee subspecies on microbial retinues. The described workflow and curated microbial database will be generally useful for microbial surveys of healthy and declining honey bees.

## Introduction

The honey bee (*Apis mellifera* L.) has ecological importance as a natural pollinator of wild flora and crops. Moreover, managed honey bees have economical importance with hive products including honey, pollen, wax, propolis, and royal jelly (Maheshwari, 2003). Recently, declines of managed colonies have been noted on many continents. Several causes of these large-scale losses have been reported, including honey bee parasites (*Varroa destructor*, *Acarapis woodi*); pathogens (*Nosema* spp. and bee viruses); pesticides, contaminated water, use of antibiotics, poor nutrition, and migratory beekeeping practices (Kevan et al., 2007; Higes et al., 2008; Naug, 2009; vanEngelsdorp et al., 2009; Bacandritsos et al., 2010; vanEngelsdorp and Meixner, 2010). The high density of individuals and the exchange of food among *A. mellifera* colony members create a favorable environment for bacterial, viral, fungal, and protist pathogens and several studies have noted an increase in diversity and infection rates of pathogens in failing bee colonies. Here we will focus on honey bee pathogens and parasites and the use of modern sequencing techniques to identify these agents in healthy and declining colonies.

Among the honey bee pathogens, viruses are of special concern. Viruses are widespread in honey bees although most often without noticeable symptoms (Ball and Bailey, 1997). Multiple viral infections have been diagnosed in many bee colonies (Chen et al., 2004). At least 18 different viruses exist in honey bees (Bailey and Ball, 1991) with six of them; Sacbrood virus (SBV), Deformed wing virus (DWV), Acute bee paralysis virus (ABPV), Black queen cell virus (BQCV), Chronic bee paralysis virus (CBPV), and Kashmir bee virus (KBV) most commonly linked to bee disease. These viruses have strong impacts on managed bee populations, pollination services, and honey production on several continents (Allen and Ball, 1996; Nordstrom et al., 1999; Ellis and Munn, 2005; De Miranda et al., 2010). DWV and ABPV have been linked to parasitic mite loads, while Chronic bee paralysis virus (CBPV) and the related Lake Sinai viruses are also widespread and tied to significant losses in honey bee colonies (Runckel et al., 2011; Ravoet et al., 2013). Sacbrood virus is the only common virus of developing bees, and this virus is not generally implicated in adult bee mortality or morbidity (Anderson and Gibbs, 1989).

Two species of Microsporidia, *Nosema apis* and *Nosema ceranae*, are widespread parasites of adult honey bees. *N. apis* is a long-standing infection agent of the European honey bee, *A. mellifera*, (Zander, 1909) that causes nosemosis, a disease with mild virulence. *N. ceranae* was first found as a parasite of the Asian honey bee, *Apis cerana* (Fries et al., 1996) although this species is now widespread throughout the range of *A. mellifera* (Fries et al., 1996, 2006; Paxton et al., 2007) arguably thanks to worldwide trade in bees (Klee et al., 2007) and perhaps pollen supplements. Honey bees can be co-infected with both *Nosema* species (Fries, 2010). Additionally, *N. ceranae* seems to be replacing *N. apis* worldwide (Klee et al., 2007). In association with *Nosema* infections, several viruses can significantly affect the apparent virulence of *Nosema* (Bailey and Ball, 1991).

Trypanosomes are a varied group of parasites that infect insects (Merzlyak et al., 2001). *Crithidia bombi*, a trypanosome that infects bumble bees, has effects on behavior (Gegear et al., 2005) and longevity during stressful conditions (Brown et al., 2003). Trypanosomatid parasites parasitize the mid- and hind-guts of their hosts (Lange and Lord, 2012) and can be widespread (Langridge and McGhee, 1967; Schmid-Hempel and Tognazzo, 2010). The current role of trypanosomes in honey bee health is not clear (Schwarz and Evans, 2013), although they have been recognized as possible correlates with bee declines in two field surveys (Runckel et al., 2011; Ravoet et al., 2013). The most common honey bee trypanosomatid currently is distinct from the species *Crithidia mellificae* described by Langridge and McGhee (1967), and has recently been named as *Lotmaria passim* (Schwarz et al., 2015).

*Spiroplasmas* are particularly virulent pathogens that are found in various environments and implicated as pathogens of plants, vertebrates and insects. They have a seasonal occurrence associated with the nectarines and surfaces of flowers (Markham and Townsend, 1981; Williamson et al., 1989). Adult honey bees are parasitized by two species of bacteria, *Spiroplasma apis* (Mouches et al., 1983) and *Spiroplasma melliferum* (Clark et al., 1985). Upon invading the hemolymph, these bacteria can cause a fatal disease called spiroplasmosis or May disease. Gut microbiota of animals living in social communities may influence their health with their functions related to nutrition, immune responses and resistance against pathogens (Dillon and Dillon, 2004; Round and Mazmanian, 2009). Surveys of 16S rDNA sequences from the honey bee indicate the presence of eight predominant species (defined as strains sharing >97% 16S rRNA identity) which account for 95% of the resident bacteria (Moran et al., 2012). These species include the beta-proteobacterium *Snodgrassella alvi* (family *Neisseriaceae*) and the gamma-proteobacterium *Gilliamella apicola* (family *Orbaceae*), the dominant Gram-negative members of the gut community, with each comprising up to 30–39% of the microbiota (Moran et al., 2012).

**Figure 1:**
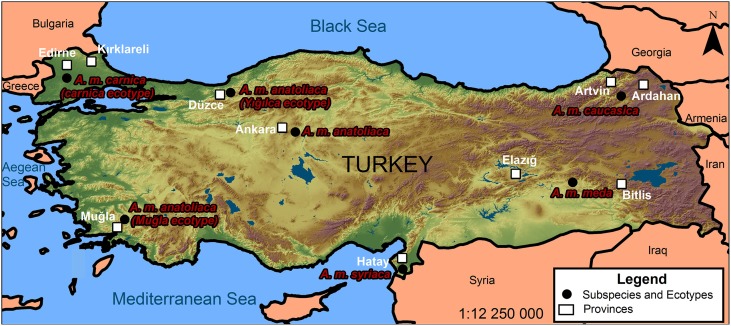
**The provinces and the distribution of honey bee subspecies in this study**.

The surveillance and discovery of novel pathogens via high-throughput sequencing can provide a relatively unbiased view of pathogens and microbes associated with insects and other arthropods (Bishop-Lilly et al., 2010; Ma et al., 2011; Vayssier-Taussat et al., 2013). Recent efforts based on these technologies have uncovered novel and unexpected taxa associated with honey bees (e.g., Cox-Foster et al., 2007; Runckel et al., 2011; Cornman et al., 2013) and have provided estimates of normal microbial levels vs. those of diseased colonies. Turkey is one of the World's most important centers of apiculture, with managed and wild populations of five subspecies of *Apis mellifera* L. including *A. m. caucasica*, *A. m. syriaca*, *A. m. anatoliaca*, *A. m. meda* and an ecotype in Thrace belonging to the carnica subspecies group which is distinctly different from the subspecies found in Anatolia (Figure 1) (Kandemir et al., 2000; Bodur et al., 2007). These subspecies cover approximately 20% of honey bee subspecies in the world. Our purpose here was to determine the regional prevalence of bacterial pathogens, viruses, fungi, parasites, protists, and symbionts and to compare their loads in areas where migratory and stationary beekeeping is practiced in Turkey. Monitoring viruses and other disease agents can help solve problems related to the health of stationary and migratory honey bee colonies and limit or avoid their spread. Although commercial migratory beekeeping practices are necessary for pollination and crop production, their effects on honey bee colony health and pathogen transmission should be addressed. We used transcriptome analyses to survey a wide range of pathogens and to detect unexpected or rare taxa. RNA-seq technology allows the precise detection of rare transcripts by mapping reads against inclusive sequence databases, reducing the repetitive effort and possible biases of conventional molecular diagnostics (Minoche et al., 2011). Identification and confirmation of select virus and pathogen loads was confirmed by quantitative RT-PCR. Our survey was aligned with the European Honey bee colony loss network (www.COLOSS.org) survey, and was conducted to determine the colony losses in many regions representing honey bee diversity in Turkey. By using metagenomic analysis, our results provide new incidence records of the virome and microbiome in search of etiologically unexpected or previously unknown agents among 10 distinct provinces in Turkey and suggest a higher viral prevalence, and increased losses, in migratory beekeeping operations. *Apis mellifera* is an economically and ecologically important model organism and identification of pathogens and other microbes can have extensive implications for current practices in apiculture and agriculture.

## Materials and methods

### Sampling

Colony loss surveys were carried out in eight regions and 158 beekeeping operations in 2010. Among those, 98 were migratory beekeepers and 60 were stationary. In 2011, surveys of 221 beekeepers from seven different regions were evaluated for this study. Samples analyzed for microbial loads comprised adult honey bees collected from field colonies in Turkey during summer and fall of 2010 and 2011. Efforts were made to collect from different regions of Turkey, and from a diverse set of beekeepers (Figure 1). Adult bees were collected from 134 colonies from different regions of Turkey from 38, 48, and 51 beekeepers in 2010-Fall, 2010-Spring, and 2011 respectfully and the samples were kept frozen until molecular diagnosis.

### RNA isolation

RNA was extracted from a pooled sample of 50 bees from each sampled colony, using an acid-phenol RNA extraction method (Evans et al., 2013).

### cDNA synthesis and real time qPCR

RNA extracts were used to generate first and second-strand cDNA's using random hexamer primers and the reverse transcriptase Superscript II® (Invitrogen™), as described in vanEngelsdorp et al. (2009). Pathogen loads were estimated using real-time quantitative-PCR (qPCR) and a Bio-Rad CFX-96™ thermocycler. Complementary DNA (cDNA) was generated from 1 μg RNA template and was amplified in a separate 20 μl final reaction volume of Sso-Fast™ SYBR® Green reaction mix (Bio-Rad™) for each diagnostic primer pair. We used published primers to survey for SBV, KBV, IAPV, DWV, ABPV, BQCV, trypanosomes (vanEngelsdorp et al., 2009), AFB (Evans et al., 2006), *Nosema ceranae* (Fries et al., 2013) and *Nosema apis* (Schwarz and Evans, 2013), *Spiroplasma apis* and *Spiroplasma melliferum* (Schwarz et al., 2014). Honey bee ribosomal protein S5 (RPS5) was used to normalize for cDNA content and to filter samples for degradation or experimental losses.

We used a thermal profile of 95°C for 30 s followed by 95°C for 5 s and 60°C for 30 s. Steps two and three were repeated for a total of 50 cycles and included plate reads for florescence during each 60°C step. Following the cycle program, products were denatured for 10 s at 95°C., reannealed and then a dissociation profile was measured between 69 and 95°C at an increment of 0.5°C to provide evidence for reaction fidelity (Evans et al., 2006). Annealing temperature of 55°C was used for the CytbSF (trypanosomatid *L. passim*) primers. Positive and negative control reactions were run on each 96-well plate. Pathogen loads (ΔΔCT) were determined as the difference between the CT of RPS5 and the CT of each target (ΔCT), scaled up from the minimal ΔCT across all samples.

### PCR purification and sequencing

RT-PCR products were selected for sequencing to confirm the identities of products indicating the trypanosome, *L. passim*, and the bacteria *S. apis* and *S. melliferum*. These PCR products were purified using QIAquick PCR purification kit according to protocol recommended by manufacturer, and then were sequenced commercially by Macrogen (Rockville, MD, USA) DNA sequence similarity with trypanosomes, *S. apis* and *S. melliferum*, was confirmed using the BLAST search tool (Altschul et al., 1990) from the U.S. National Institutes of Health and searches against the National Center for Biotechnology Information (NCBI) *nr* database.

### High throughput sequencing and data analysis

High throughput sequencing was performed with the pooled samples from stationary colonies of each region and all RNA samples were pooled before sequencing (*n* = 6 ILLUMINA RNA libraries). Libraries were run on paired-end 100-cycle reactions and flow cells using Illumina Hi-Seq 2000 machines at the University of Maryland Institute of Genome Sciences. Sequences for all six libraries have been deposited at the US National Institutes of Health NCBI “Honey Bee Disease Database” Bioproject (PRJNA52851).

**Figure 2:**
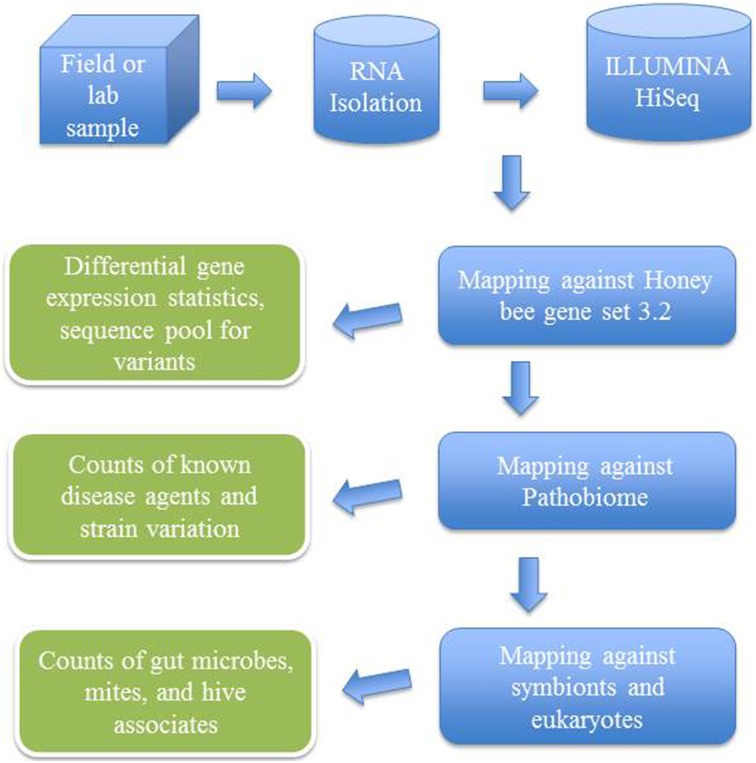
**Flow chart showing analytical steps for mapping ILLUMINA RNA-seq reads against an annotated bee microbial dataset**.

After trimming and quality control of the generated sequences, transcriptomic analysis was done with the support of CLC Genomics Workbench 7.0.3 (CLC Bio, Aarhus, Denmark) by mapping sequencing reads and counting and distributing the reads across genes and transcripts based on annotated reference genes (Figure 2). During the alignment, two mismatches were allowed with deletion cost of three, insertion cost of three, length fraction of 0.8, similarity fraction of 0.8 and maximum 10 hits for a read. For statistical analyses, proportions-based tests were used for the comparison of the counts by considering the proportions that they comprise of the total sum of counts in each sample. Multi-group comparison was done by weighted *t*-type test statistic Baggerly's test (Baggerly et al., 2003). FDR (False Discovery Rate) corrected *p*-values were calculated according to the methods of Benjamini and Hochberg (1995) to determine the statistical significance of the pathogen load.

Real time q-PCR data statistics were performed by using JMP^™^ (SAS Institute, Cary, NC, USA, v.9). A One-Way analysis of variance was used to test differences between group means. The total variability in the response was divided into two parts; within-group variability and between-group variability. The differences between the group means were considered to be significant if the between-group variability was broader relative to the within-group variability. Multiple comparisons of group means were done by using pooled variance estimates for these means. Student's *t*-tests were computed for each pair of group levels and individual pairwise comparisons. Matrix of correlation coefficients that summarized the strength of the linear relationships between each pair of response variables was calculated and Pearson product-moment correlations for each pair of variables were listed. Correlations and the significance probabilities were calculated by the pairwise deletion method and the count values differed if any pair had a missing value for either variable.

## Results

### RT-qPCR results

Kashmir bee virus (KBV), Israeli acute paralysis virus (IAPV), American foulbrood (AFB) and Sacbrood virus (SBV) were not detected by PCR in any of the samples, nor was the microsporidian pathogen *N. apis* (except for only three colonies in 2011) by RT-PCR. Present in these samples were Acute bee paralysis virus (ABPV), Deformed wing virus (DWV), Black queen cell virus (BQCV), and *N. ceranae*. Mixed infections of ABPV, DWV, BQCV, and *N. ceranae* were detected (Tables 1, 2).

**Table 1:** **The results of pathogen analysis of honey bee samples collected from different regions of Turkey**.

**Sampling date**	**Locations**	**Pathogens**
2010–2011	Muğla	ABPV, BQCV, DWV, *Nosema ceranae*, Trypanosomes
2010–2011	Hatay	ABPV, BQCV, DWV, *Nosema ceranae*, Trypanosomes
2010	Ankara	ABPV, BQCV, DWV, *Nosema ceranae*, Trypanosomes
2011	Yığılca	ABPV, BQCV, DWV, *Nosema ceranae*, Trypanosomes
2010–2011	Kırklareli	ABPV^*^, BQCV, DWV, *Nosema ceranae*, Trypanosomes
2010–2011	Ardahan	ABPV, BQCV, DWV, Nosema ceranae^*^, Trypanosomes
2010–2011	Artvin	ABPV, BQCV, DWV, Nosema ceranae^*^, Trypanosomes
2010	Bitlis	ABPV, BQCV, DWV, *Nosema ceranae*, Trypanosomes
2010	Edirne	BQCV, DWV, Trypanosomes
2010	Elazığ	BQCV, DWV, Trypanosomes

* *Detected among samples of 2011 only*.

**Table 2:** **Normalized pathogen loads across sites and between stationary and migratory beekeepers for 2010 and 2011**.

	**DWV**		**ABPV**		**BQCV**		***N. ceranae***		**TRYP**	
	**2010**	**2011**	**2010**	**2011**	**2010**	**2011**	**2010**	**2011**	**2010**	**2011**
**STATIONARY BEEKEEPERS**										
All sites	4.99	11.59	1.34	13.87	5.36	6.31	0.19	10.97	8.82	14.36
Muğla	7.02	12.57	0.37	11.6	5.83	3.94	0.42	3.81	ND	11.74
Hatay	10.99	15.16	3.42	8.69	8.86	5.99	ND	ND	12.36	1.91
Yığılca	/	17.55	/	19.40	/	ND	/	9.48	/	20.83
Kırklareli	3.88	9.15	ND	12.37	4.26	5.02	0.91	15.20	0.94	7.04
Ardahan	7.56	9.00	2.57	17.80	ND	13.00	ND	13.67	ND	16.28
Artvin	ND	6.13	2.26	13.37	5.78	9.92	ND	23.67	24.90	28.37
Ankara	4.31	/	0.78	/	11.64	/	ND	/	21.59	/
Edirne	1.14	/	ND	/	1.15	/	ND	/	1.92	/
**MIGRATORY BEEKEEPERS**										
All sites	4.81	17.08	6.32	18.09	8.13	5.99	1.54	4.69	13.92	15.89
Muğla	6.62	17.77	3.60	15.42	8.64	6.52	2.25	5.34	23.04	18.54
Hatay	8.21	16.38	5.05	20.75	10.32	5.45	2.69	4.04	14.77	13.24
Ardahan	ND	/	4.63	/	13.16	/	ND	/	26.24	/
Ankara	ND	/	4.91	/	7.28	/	0.71	/	2.15	/
Bitlis	11.56	/	19.72	/	6.33	/	3.57	/	12.92	/
Elazig	2.44	/	ND	/	3.05	/	ND	/	4.38	/

*Abundances are log-based-two scale so every increase by one number reflects a doubling of pathogen RNA. ND, Not detected;/, Sampling was not done*.

The distribution of bee pathogens was significantly different among provinces and with beekeeping practices in 2010 (Table 3 in Supplementary Material). Generally, DWV loads were higher in Bitlis, Hatay, Muğla, and Ardahan than in other regions. ABPV was the most common virus in Bitlis and was especially high in the samples of migratory beekeepers. Among provinces that were sampled, BQCV loads were lowest in Edirne and highest in colonies of migratory beekeepers sampled in Ardahan, Hatay, and Muğla (Table 2). In 2011, DWV, BQCV, and *N. ceranae* loads were significantly different among the regions (Table 3 in Supplementary Material). DWV loads were higher in Muğla, Hatay, and Yığılca. BQCV levels were high in Ardahan and Artvin and was not detected at all in Yığılca. *N. ceranae* was more frequent among Artvin, Ardahan, and Kırklareli but not detected among stationary colonies of Hatay (Table 2). ABPV occurrence was the highest in samples from stationary apiaries of Yığılca and Ardahan and samples from migratory ones of Hatay and Muğla. The lowest level was in the stationary colonies of Hatay. ABPV loads showed difference between Yığılca-Kırklareli and Yığılca-Muğla (*p* = 0.0398 and *p* = 0.0478).

ABPV loads in Muğla and Ankara, BQCV loads in Ardahan, Muğla, and *N. ceranae* loads in Hatay were significantly higher in the samples of migratory beekeepers than the samples of stationary beekeepers in 2010. Among 2011 samples, the pathogen loads of migratory colonies were also higher in Muğla and Hatay but the results were not significant (Table 3 in Supplementary Material).

In 2010, *N. ceranae* was widespread in migratory colonies of Muğla, Hatay, and Bitlis and less frequent in samples from Ankara and Kırklareli. *N. ceranae* was not found in samples of beekeepers from Edirne, Artvin, Ardahan, and Elazığ in 2010 (Table 2). In contrast, *N. ceranae* showed high incidence in all sites except for the stationary colonies of Hatay in 2011 (Table 2). *N. ceranae* loads were higher among migratory colonies than stationary ones in both years. *N. apis* was not observed among the samples of 2010 and most of the samples of 2011 although the analysis was repeated twice. In 2011, *N. apis* was detected in three stationary colonies in total, from three beekeepers in Muğla-Bodrum, Düzce-Nas, and Ardahan-Posof.

Trypanosome loads were significantly different among regions in both years. Trypanosome abundance was higher within the samples of migratory beekeepers than those of stationary ones (Table 3 in Supplementary Material). Ten/ten positive samples were confirmed by DNA sequencing as reflecting the 28S rRNA locus of trypanosomes. In general trypanosome levels were higher among samples from Muğla, Ankara, Artvin and Ardahan compared to the samples from Kırklareli, Edirne and Elazığ, for 2010. In 2011, levels were especially high in samples from Artvin, Yığılca, and Muğla, and low in samples of stationary colonies in Hatay and Kırklareli (Table 2). Seasonal variation was observed among samples collected in 2010 (ANOVA *p* = 0.0023). Specifically, trypanosome loads were highest in spring as opposed to fall. In Ardahan and Hatay, trypanosomes were not detected among any of the fall samples. Among the samples with trypanosome infection, thirteen 2010 samples for which products were assayed by DNA sequencing were all confirmed as being part of the “SF” strain of *L. passim* (identified as *C. mellificae*) (Runckel et al., 2011) and 10 were positive among 51 samples in 2011, based on qPCR and melt-curve analyses (seven products were confirmed by DNA sequencing). four of the “SF” positive samples were from Muğla, six from Hatay, eight from Artvin-Ardahan, four from Yığılca, and one from Elazığ. GAPDH sequence analyses from Artvin and Yığılca all matched *L*. passim, haplotype A (Morimoto et al., 2013), indicating that this haplotype was more common among the samples when compared with haplotype B.

*S. melliferum* was detected for two samples in Artvin province in 2010 while 13 samples were positive for Artvin, Ardahan, Muğla, Yığılca, and Kırklareli during 2011. Six of the positive samples were confirmed by DNA sequencing. *S. apis* was detected in only two samples from Muğla and Elazığ in 2010 and in one sample from Yığılca in 2011.

Dual infections of DWV and BQCV were detected in both migratory and stationary beekeepers in 2010 (50–100% of honey bees in six provinces sampled). Dual infections with these two viruses were less commonly observed in 2011 (18–40% of honey bees in four of the provinces sampled). Triple infections of ABPV, BQCV, and DWV were seen in three regions (50–100% of honeybee samples) in 2010, whereas occurred less (18–40% of honey bee samples) at four regions in 2011. Among 2010 samples, *N. ceranae* loads were correlated with DWV loads (*r* = 0.25, *p* = 0.0186). ABPV loads were correlated with both DWV (*r* = 0.40, *p* = 0.0001) and *N. ceranae* (*r* = 0.35, *p* = 0.0009). BQCV correlated with DWV within samples from Ankara, Bitlis, Edirne, and Muğla. In Bitlis, ABPV levels were positively correlated with DWV (*r* = 0.89, *p* = 0.0061). In Hatay, BQCV correlated with *N*. *ceranae* (*r* = 0.47, *p* = 0.0443). Among 2011 samples ABPV showed correlations with DWV (*r* = 0.29, *p* = 0.0381) and in some regions with *N. ceranae*. A positive correlation was observed between trypanosome load and *N. ceranae* (*r* = 0.28, *p* = 0.0083) and between trypanosomes and BQCV (*r* = 0.59, *p* < 0.0001).

**Figure 3:**
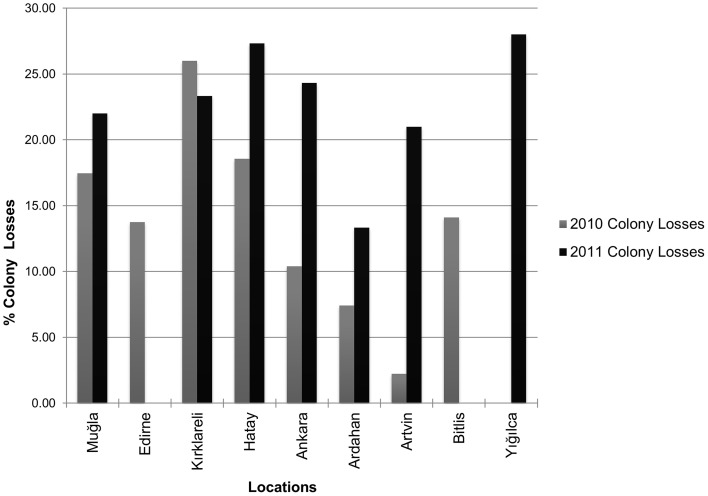
**Percent colony losses of surveyed beekeepers by location in 2010 and 2011**.

According to the survey results, there was an increase in colony losses in 2011 when compared with 2010 losses (Figure 3). Kırklareli, Muğla, Hatay, and Ankara had relatively higher colony losses in both years while Ardahan and Artvin tended to have lower losses than the other provinces. High colony losses were also observed in Edirne and Bitlis in 2010 and especially in Yığılca in 2011. Average colony losses of migratory beekeepers were significantly higher than those of the stationary beekeepers.

### Read mapping results

**Figure 4:**
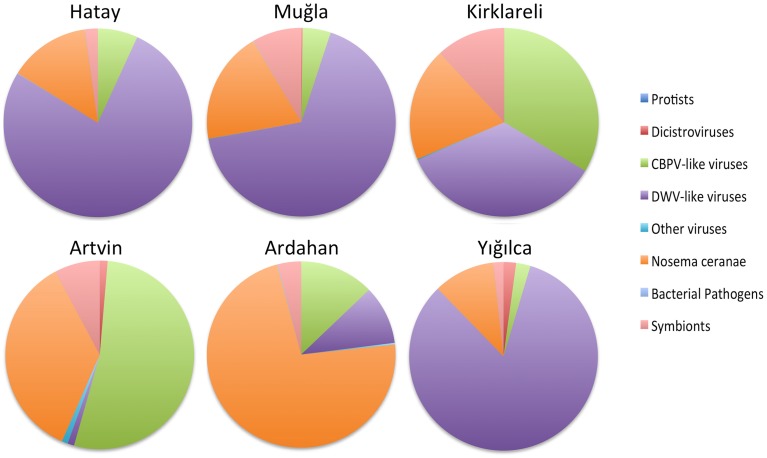
**Relative microbial loads across the six regions, as inferred by RNA-Seq matches**. Taxa are binned by taxonomic group.

DWV reads were detected in sequenced RNA libraries from all of the regions (Figure 4 and Table 4 in Supplementary Material), although they were most highly represented in Hatay, Yığılca, and Muğla, matching the results from real time q-PCR. We found strong evidence for *Varroa destructor* virus-1 as well, a first for these regions. Overall, the DWV/VDV group of iflaviruses was the predominant viral taxon (Figure 4). RNA sequences of BQCV were highest in the Ardahan region, although BQCV reads were also abundant in other provinces. Consistent with the fact that BQCV was not detected in Yığılca by RT-qPCR very few reads were found in this province during RNA sequencing According to the sequence analysis, ABPV contigs were common in most regions. Consistent with RT-PCR results, they were most frequently observed in Yığılca and Ardahan. Very few CBPV reads were found in Kirklareli province and this virus was not detected or was very few in other regions. In contrast, the related Lake Sinai viruses were highly represented both in diversity across this large clade, and in abundance. IAPV and KBV were notably rare or were not present across all regions. Sacbrood virus was similarly rare, although 1885 SBV reads were detected in Ardahan region. SBPV reads (Slow Bee Paralysis Virus) were not observed in any of the regions (Table 4 in Supplementary Material).

*Nosema ceranae* was highly prevalent in our RNA-seq analysis. *N. ceranae* was especially common in Artvin province, again matching our RT-qPCR analyses. *N. ceranae* was not detected in stationary colonies of Hatay province but it was evident there by RNA-seq analysis. We found minimal, if any reads matching *N. apis* in the RNASeq data, those present were largely the result of regions with high sequence similarity to *N. ceranae*. Reads for the betaproteobacterium, *Snodgrassella alvi* were common among all of the provinces with highest frequency in Kırklareli, Muğla, Yığılca, and Ardahan. The gamma-proteobacterium, *Gilliamella apicola* was prevalent in all regions, while the related *Frischella perrara* was rare (Table 4 in Supplementary Material). Consistent with both RT-qPCR and DNA sequencing, the presence of *Spiroplasma melliferum* was confirmed in most of the regions. The abundance of congener *S. apis* was confirmed with the RNA-seq analysis despite being missed by PCR, yet *S. apis* remained the minor of the two species.

## Discussion

### Evaluation of real time q-PCR results

The surveyed regions comprise important centers of beekeeping in Turkey. Kashmir Bee Virus (KBV) and Israel Acute Bee Paralysis Virus (IAPV) were not detected in sampled colonies by RT-qPCR, while the related virus Acute Bee Paralysis Virus (ABPV) was abundant in most of the samples and was present in the regions where colony declines were observed. Historically, KBV has been found most frequently in the United States and Australia (Allen and Ball, 1996) and this virus is thought to be an exotic bee virus in central Europe (Berenyi et al., 2006). Our discovery of just one of these three species could reflect climatic and environmental conditions (Anderson, 1991) or differential abilities of these viruses to infect different honey bee subspecies.

DWV was detected in both migratory and stationary operations in all of the regions, consistent with the high prevalence of this virus worldwide (Tentcheva et al., 2004; Berenyi et al., 2006; Kukielka et al., 2008; Nielsen et al., 2008; Welch et al., 2009). The presence of DWV has been reported in colonies of *Apis mellifera* L. in Ordu province of Turkey (Gülmez et al., 2009). BQCV was the second most widespread bee virus in our study. BQCV is also the second-most prevalent virus in honey bee colonies in Asia and Europe (Tentcheva et al., 2004) and its presence was confirmed in many studies (Berenyi et al., 2006; Kukielka et al., 2008; Welch et al., 2009; Choe et al., 2012). BQCV was previously reported in 21.42% of 28 bee samples from six provinces of the Black Sea Region in Turkey (Gümüşova et al., 2010). BQCV was more prevalent in migratory bee colonies, consistent with the results found for migratory bees sampled in the U.S. (Welch et al., 2009).

In most of the regions we studied, BQCV and DWV were found together more often than expected by chance. This result is consistent with the findings in a previous study of virus infections (Chen et al., 2004) for which these distantly related viruses were found coinfecting honey bees at a high frequency. Triple infections of ABPV, BQCV, and DWV were detected in some colonies. Nielsen et al. (2008) also found a high incidence of dual and triple infections. According to Chen et al. (2004), 50% of colonies in the USA had dual infections while 7% had triple infections. Simultaneous multiple infections were also common in Austria (Berenyi et al., 2006), Southwest England (Baker and Schroeder, 2008), Brazil (Teixeira et al., 2008), Jordan (Haddad et al., 2008), France (Tentcheva et al., 2004), and Hungary (Forgach et al., 2008).

In Turkey, the presence of *N. apis* was confirmed earlier (Aydin et al., 2005; Muz et al., 2010; Whitaker et al., 2010). Using qPCR, we detected *N. apis* in only three colonies, indicating the replacement of *N. apis* by *N. ceranae* in many regions included in this study. Worldwide, *N. ceranae* is now far more common than *N. apis* (Chen et al., 2009; Valera et al., 2011; Yoshiyama and Kimura, 2011).

In 2006, *N. ceranae* was found in the provinces of Artvin, Hatay, and Muğla (Whitaker et al., 2010). Collapsed colonies from the Hatay overwintering region and Southeastern Marmara were found to show infections of *N. ceranae* (Muz et al., 2010). In our 2010 samples, *N. ceranae* was observed in Bitlis, Hatay, and Muğla and not in Edirne, Artvin, Ardahan, and Elazığ in 2010. Interestingly, *N. ceranae* was widely common for all of the regions involved in the study in 2011. Detection of *N. ceranae* among 2011 samples of Ardahan-Artvin might be the reason for the increase in colony losses in these provinces in 2011. The incidence of *N. ceranae* in Kırklareli province in 2011 was much more than the previous year. *N. ceranae* loads of samples from migratory beekeepers were significantly higher than the stationary ones and *N. ceranae* loads were correlated the viruses ABPV, BQCV and DWV. We propose that the infectivity of *N. ceranae* expands with migratory beekeeping activities and in association with different viruses and trypanosomatids. In July, the Thrace region is a highly frequented location by migratory beekeepers seeking to harvest sunflower honey. It is thought that these beekeepers in this region had colony losses because of the application of pesticides rather than honey bee disease factors. Hatay and Ankara are also important locations for migratory beekeepers. Caucasian bees which are used by most of the migratory beekeepers, are raised in Ardahan and Artvin. Bitlis also suffered high colony losses in 2010, combined with high disease loads among samples of migratory beekeepers. In contrast, while honeybee samples in Yığılca were derived from stationary colonies, high colony losses and high pathogen loads were observed. Probably some other factors were contributing to colony losses in this province. All five viruses and pathogens were also detected in Muğla, the center of migratory beekeeping. These results could reflect the differences in pathogen exposure of local and migratory colonies, varying resistance levels, or perhaps a differential ability to handle stress. Genetic impacts of migratory beekeeping has become an important concern in Turkey and elsewhere. Population structure can be disturbed with hybridization, leading to a loss of regionally adaptive traits and perhaps decreasing colony fitness.

Trypanosomes were highly prevalent in our samples. Trypanosomes have also been reported in Australia (Langridge and McGhee, 1967), China (Yang et al., 2013), France (Dainat et al., 2012), Japan (Morimoto et al., 2013), Switzerland (Schmid-Hempel and Tognazzo, 2010), USA (vanEngelsdorp et al., 2009; Runckel et al., 2011), and Spain (Orantes-Bermejo, 1999). Trypanosome levels were higher in summer samples than the fall samples in our samples. In southern Spain, this parasite appears in July and August (Orantes-Bermejo, 1999) and *L. passim* (recently named as the primary trypanosomatid in lieu of *C. mellificae*, Schwarz et al., 2015) was common in summer colonies in Belgium (Ravoet et al., 2013). These findings are in contrast with the results of Runckel et al. (2011) which show a peak in *L. passim* levels in January in U.S. colonies.

Synergistic effects can make colonies more vulnerable to other pathogens (Cornman et al., 2012) and we noted a positive correlation between *L. passim* and *N. ceranae* prevalence. Similarly, this relationship was documented in field surveys from the U.S. (Runckel et al., 2011) and the co-occurence of *L. passim* and *N. ceranae* in summer was tied to higher colony mortality in Belgium (Ravoet et al., 2013). Complex dynamic immune responses of honey bees to both *Nosema* and trypanosomatids were recently reported (Schwarz and Evans, 2013) and it will be interesting to test further for mechanistic explanations for any synergisms between these parasites.

### Metagenomic sequencing

As in prior studies, iflaviruses related to Deformed wing virus (DWV) were especially prevalent. While DWV amounts were higher, reads for the closely related *Varroa destructor*-1 virus (VDV-1) were fairly abundant in Hatay, Yığılca, and Muğla. CBPV reads were relatively rare in most of the regions and were not detected in Yığılca and Ardahan. CBPV was reported in seven of 28 (25%) samples from six provinces of Black Sea region in Turkey (Gümüşova et al., 2010). CBPV was detected in four of 96 apiaries in survey study of Denmark, in 73% of the samples from Greek and 9% of apiaries in China (Nielsen et al., 2008; Bacandritsos et al., 2010; Ai et al., 2012). A recently described relative of CBPV, Lake Sinai virus, was prevalent in all regions, second only to the DWV and VDV group. In fact, this group was the most prevalent virus in Kirklareli and Ardahan provinces. The pathogenic or epidemiological significance of Lake Sinai viruses are not well-known. The LSV species complex is diverse, with members sharing between 70 and 99% sequence identity, hence this group is often missed in screenings based on PCR. LSV4 appears to be especially abundant, along with LSV1 and LSV2. There was significant variation across the regions in the specific lineages seen for this group. LSV2 was the most abundant single component of the honey bee microbiome in the study of Runckel et al. (2011). The presence of LSV was also confirmed in honey bees from Spain by high-throughput sequencing (Granberg et al., 2013). A new fourth strain of Lake Sinai Virus (LSV) was identified in the study of Ravoet et al. (2013).

In this study, despite tens of millions of microbial gene reads, few reads matching IAPV, KBV, and SBV were seen. IAPV, KBV, and SBV were not detected using RT-qPCR, indicating that RNA-seq sensitivity was higher than that of qPCR, or that current qPCR primers for this group must be redesigned to capture all strains. Given the RNA-Seq data, we can consider that these pathogens are quite rare in our samples. KBV has been absent in some other European surveys (e.g., Berenyi et al., 2006; Forgach et al., 2008) and rare in surveys in France, Denmark, and United Kingdom (Tentcheva et al., 2004; Ward et al., 2007; Nielsen et al., 2008). IAPV is prevalent in the Middle East and Australia (Maori et al., 2007; Palacios et al., 2008) and this species was reported in 71 samples containing 10 bees; each from 20 provinces in Turkey (Ozkirim and Schiesser, 2013). In this study IAPV wasn't confirmed by RT-qPCR in our samples and RNA sequence analysis revealed that this virus was not present in Muğla and Hatay and was incredibly rare among samples from Artvin, Yığılca, and Ardahan. SBV was similarly scarce in this study. This conflicts with results of Tentcheva et al. (2004) but overlaps with the results of Baker and Schroeder (2008) and Forgach et al. (2008). As with most surveys, we examined only adult honey bees. SBV causes a fatal disease in honey bee larvae (Bailey, 1975), thus brood samples could provide more evidence for its prevalence.

*Apis mellifera* filamentous virus (AmFV) was not detected within our samples, nor was VdMLV (Varroa Macula-like virus). A plant-pathogenic RNA virus, tobacco ringspot virus (TRSV) was not detected in any of the RNA samples in our study.

Recent culture-independent studies reported eight bacterial phylotypes inhabiting the gut of the honey bee, *Apis mellifera* from several continents (Jeyaprakash et al., 2003; Mohr and Tebbe, 2006; Babendreier et al., 2007; Cox-Foster et al., 2007; Olofsson and Vasquez, 2008). Colonies founded by swarms, interactions within the colony, intercolony interactions like robbing food in neighboring hives and mixing of colonies by beekeepers all might affect the gut microbiota (Engel et al., 2012). In our study, unique gene reads of *Snodgrassella alvi* and *Gilliamella apicola* were highly represented and showed differences among provinces because of geographic, environmental and subspecies differences of hosts. The differences in social behaviors of the subspecies, the dietary sources and exposure to varying pathogens and pesticides might influence the abundance of these bacteria among regions. One gamma-proteobacterial member of the gut microbiota *Candidatus Schmidhempelia bombi*, was present in 90% of bumble bee individuals in the study of Martinson et al. (2014). This symbiont was prevalent in all of our surveyed locations and widely represented in Hatay, Yığılca, and Muğla. The recently described gamma-proteobacterium *Frischella perrara* (Engel et al., 2013) was present in our study albeit at low levels.

*Lactobacillales* symbionts have been proposed as actors in both nutrition and parasite defenses of honey bees. *Lactobacillales* stimulate the innate immune system, arguably increasing honey bee defenses against disease agents (Evans and Lopez, 2004). Along with their impacts on immunity, the microbial symbionts have been proposed to nutritionally compete with pathogens by occupying the available niches (Crotti et al., 2012). In this study, very low number of 16S *Lactobacillus* reads were observed among regions. Overall bacterial loads were especially high in Hatay, Yığılca, and Artvin. The prevalence of the bacterial pathogens *S. apis* and *S. melliferum* is low among Belgian honey bee colonies (Ravoet et al., 2013), and these bacteria are present only seasonally in North and South America (Runckel et al., 2011; Schwarz et al., 2014). We found low *Spiroplasma* levels, with highest incidence in Artvin province. *S. melliferum* was the more common species, matching results from the Americas (Schwarz et al., 2014). The bacterial brood diseases European Foulbrood (EFB) caused by the bacterium *Melissococcus plutonius* (Bailey et al., 1983) and American Foulbrood (AFB) caused by the bacterium *Paenibacillus larvae* (Genersch et al., 2006) are globally important diseases of honey bees. *P. larvae* reads were not common among our samples. Similarly, *M. plutonius* was not prevalent among the regions but was more ubiquitous in Hatay, Muğla, and Yığılca. Environmental conditions at these sites can be conductive for the expression of the disease. Like AFB, EFB transmission is also linked to larval immune responses (Evans, 2004), hygienic behavior (Spivak and Reuter, 2001) as well as interaction between *M. plutonius* and the intestinal microbiota of the honey bee larvae (Gilliam, 1997; Olofsson and Vasquez, 2008), nutritional and stress conditions, weather and geography (Bailey, 1961).

Sequences for *Ascosphaera apis*, the causative agent for Chalkbrood disease, were generally rare, with the highest incidence from samples of in Hatay province. Similarly, neogregarines (nominally *Apicystis bombi*) persisted at extremely low levels among regions in this study. Among arthropod parasites of honey bees, we found no genetic evidence for the presence of the tarsonemid tracheal mite, *Acarapis woodi* within our samples. The Asian parasitic mite *Tropilaelaps* is considered more dangerous to *A. mellifera* than the parasitic mite *Varroa destructor* (Rath et al., 1995), and this mite is worthy of screening. Our deep sequencing analysis showed no sign of *Tropilaelaps*. The presence of phorid flies (*Apocephalus borealis*) in the study of Ravoet et al. (2013) proves their existence in Europe, but our deep sequencing did not reveal signs of this parasite.

In conclusion, we screened bee-derived RNA against the most complete sequence set for honey bee associates used to date. We assessed levels of known and novel parasites, pathogens, and symbionts. We present quantitative data for bacterial pathogens (*Melissococcus plutonius*, *Paenibacillus larvae*, *S. apis*, *S. melliferum*), protists (*Apicytis*, trypanosomatids), viruses (Lake Sinai virus, Chronic bee paralysis virus, Deformed wing virus, *Varroa destructor* virus, Sacbrood, and Dicistroviruses), symbionts (*Candidatus Schmidhempelia bombi*, *Frischella perrara*, *Snodgrassella alvi*, *Gilliamella apicola*, *Lactobacillus* spp., *Acetobacteracea*), microsporidia and fungi in *A. mellifera* colonies in distinct regions of Turkey. The presence of KBV, SBPV, Tobacco ringspot virus, VdMLV (Varroa Macula like), *Acarapis* spp., *Tropilaelaps claerae* and *Apocephalus* (phorid fly) were also examined. As in other countries, bee viruses were correlated with colony losses in Turkey. In comparison with 2010, the increase in pathogen loads in 2011 might be a factor for increased colony losses observed in this study. It seems likely that migratory beekeeping practices enable the spread of disease factors among honey bees in places where they visit and causing an important threat to the honey bee colonies. In addition, the impacts of parasites and pathogens varies between regions, perhaps reflecting different honey bee genetic traits. Migratory beekeeping was correlated with both higher disease loads and a potential risk of dispersing regional parasites and pathogens across the country. This practice also allows for greater gene flow between migratory honey bee populations and local populations. Current diversity and local genetic structure can be preserved with selection strategies and establishing broad areas of isolation to reduce the risks of migratory beekeeping practices. While experimental work and longitudinal analyses will be needed to confirm causes of bee declines, our analyses, reference sequences, and strategy will help reduce the set of likely causes.

### Conflict of interest statement

The authors declare that the research was conducted in the absence of any commercial or financial relationships that could be construed as a potential conflict of interest.
