# Supplementary material for: Metatranscriptomic analyses of honey bee colonies
Source: Front Genet. 2015 Mar 19;6:100. doi: 10.3389/fgene.2015.00100 (PMC4365734; doi:10.3389/fgene.2015.00100)
Supplement: Supplementary file 1 [file Table3.DOCX]

**Metatranscriptomic analyses of honey bee colonies**

**C. Özge Tozkar_1_^1*^, Meral Kence_2_^1^ , Aykut Kence_3_^1#^, Qiang Huang_4_^2^, Jay D. Evans_5_^2*^**

^1^ Ecological Genetics Laboratory, Middle East Technical University, Department of Biological Sciences, Ankara, Turkey

^2^ USDA-ARS Bee Research Laboratory, Beltsville, Maryland, USA

**^#^** deceased

***Correspondence**: ^*^Jay D. Evans, USDA-ARS Bee Research Laboratory, 10300 Baltimore Avenue Bldg 306 BARC-E, Beltsville, Maryland, 20705-0000, USA. jay.evans@ars.usda.gov

^*^C. Özge Tozkar, Ecological Genetics Laboratory, Department of Biological Sciences, Middle East Technical University, Dumlupınar Bulvarı No:1, Cankaya, Ankara, 06800, Turkey.

tozkar@metu.edu.tr

**Key Words: *Apis mellifera*, pollination, Colony Collapse Disorder, RNA Sequencing, bioinformatics, honey bee viruses, trypanosomes**

**Table 3** One-way Anova results among provinces, in all regions with beekeeping type and in each province according to the beekeeping type in 2010 and 2011. ‘ND’ not detected, ‘/’ sampling was not done, ‘*’ significant.

| **Year** | **2010** | | | | | **2011** | | | | |
| --- | --- | --- | --- | --- | --- | --- | --- | --- | --- | --- |
| **Pathogens** | **DWV** | **ABPV** | **BQCV** | ***N.ceranae*** | **TRYP** | **DWV** | **ABPV** | **BQCV** | ***N.ceranae*** | **TRYP** |
| **Among provinces** | <0.0001* | <0.0001* | <0.0001* | 0.0153* | 0.0013* | 0.0349* | 0.1954 | 0.0329* | 0.0004* | <0.0001* |
| **With Beekeeping** | 0.6805 | <0.0001* | <0.0001* | 0.0005* | 0.0001* | 0.4168 | 0.0512 | 0.7546 | 0.4632 | 0.0271* |
| **Muğla** | 0.8054 | 0.0004* | 0.0252* | 0.0799 | <0.0001* | 0.3874 | 0.4789 | 0.6602 | 0.7764 | 0.1436 |
| **Hatay** | 0.0013* | 0.2757 | 0.2805 | 0.0065* | 0.6752 | 0.8698 | 0.0652 | 0.9351 | 0.2199 | 0.0024* |
| **Ardahan** | 0.0073* | 0.115 | <0.0001* | ND | <0.0001* | / | / | / | / | / |
| **Ankara** | 0.0009* | 0.0273* | 0.0009* | 0.2162 | <0.0001* | / | / | / | / | / |
