# Supplementary material for: Metatranscriptomic analyses of honey bee colonies
Source: Front Genet. 2015 Mar 19;6:100. doi: 10.3389/fgene.2015.00100 (PMC4365734; doi:10.3389/fgene.2015.00100)
Supplement: Supplementary file 2 [file Table4.DOCX]

**Metatranscriptomic analyses of honey bee colonies**

**C. Özge Tozkar_1_^1*^, Meral Kence_2_^1^ , Aykut Kence_3_^1#^, Qiang Huang_4_^2^, Jay D. Evans_5_^2*^**

^1^ Ecological Genetics Laboratory, Middle East Technical University, Department of Biological Sciences, Ankara, Turkey

^2^ USDA-ARS Bee Research Laboratory, Beltsville, Maryland, USA

**^#^** deceased

***Correspondence**: ^*^Jay D. Evans, USDA-ARS Bee Research Laboratory, 10300 Baltimore Avenue Bldg 306 BARC-E, Beltsville, Maryland, 20705-0000, USA. jay.evans@ars.usda.gov

^*^C. Özge Tozkar, Ecological Genetics Laboratory, Department of Biological Sciences, Middle East Technical University, Dumlupınar Bulvarı No:1, Cankaya, Ankara, 06800, Turkey.

tozkar@metu.edu.tr

**Key Words: *Apis mellifera*, pollination, Colony Collapse Disorder, RNA Sequencing, bioinformatics, honey bee viruses, trypanosomes**

**Table 4** Normalized read counts for hits to parasites, pathogens and symbionts in RNA Sequencing efforts for six sites in Turkey

| **Target** | **Hatay** | **Mugla** | **Kirklareli** | **Artvin** | **Ardahan** | **Yigilca** |
| --- | --- | --- | --- | --- | --- | --- |
| **Bacterial Pathogens** |  |  |  |  |  |  |
| *Melissococcus_plutonius* | 2131 | 1324 | 153 | 490 | 83 | 1172 |
| *Paenibacillus larvae* | 67 | 40 | 6 | 16 | 15 | 33 |
| *Spiroplasma apis* | 56 | 20 | 5 | 107 | 2 | 22 |
| *Spiroplasma melliferum* | 59 | 20 | 4 | 1482 | 20 | 24 |
|  |  |  |  |  |  |  |
| **Protists** |  |  |  |  |  |  |
| *Apicystis (*Neogregarine) | 5 | 6 | 4 | 2 | 4 | 3 |
| *Crithidia mellificae* 30254 | 0 | 0 | 0 | 2 | 0 | 1 |
| *Crithidia sp. Haplotype A* | 0 | 1 | 0 | 4 | 0 | 3 |
| *Crithidia sp. Haplotype B* | 0 | 0 | 0 | 0 | 0 | 0 |
| *Crithidia sp*. GAPDH | 0 | 0 | 0 | 17 | 0 | 30 |
|  |  |  |  |  |  |  |
| **Virus** |  |  |  |  |  |  |
| ABPV | 63 | 5587 | 4 | 76 | 22506 | 144296 |
| BQCV | 2164 | 1614 | 2655 | 4954 | 15445 | 350 |
| CBPV | 21 | 3 | 1967 | 3 | 0 | 1 |
| DWV | 2317199 | 306436 | 36061 | 5520 | 7410 | 854713 |
| *Apis* filamentous virus | 0 | 0 | 0 | 2 | 1 | 0 |
| IAPV | 0 | 0 | 0 | 3 | 45 | 4 |
| KBV | 0 | 0 | 0 | 0 | 0 | 0 |
| LSV1Genome | 129807 | 47613 | 247873 | 124359 | 195420 | 53052 |
| LSV2 Genome | 84554 | 30851 | 139260 | 4941 | 437186 | 67900 |
| LSV strain 324 | 4067 | 10 | 576 | 16 | 1329 | 3619 |
| LSV strain 55 | 530 | 448 | 7762 | 4 | 51526 | 1548 |
| LSV strain 56 | 3331 | 1044 | 9273 | 8 | 32314 | 2619 |
| LSV strain Av | 1 | 1 | 0 | 9 | 22 | 246 |
| LSV strain e101 | 5179 | 3835 | 4993 | 16 | 25402 | 4122 |
| LSV strain e31 | 2 | 0 | 781 | 0 | 81 | 259 |
| LSV strain e35 | 76 | 0 | 135 | 1 | 1207 | 683 |
| LSV strain Navarra | 11386 | 3692 | 13352 | 13712 | 17682 | 9934 |
| LSV3 | 4880 | 115 | 4405 | 107 | 21367 | 3980 |
| LSV4 | 62466 | 20842 | 133211 | 85805 | 88629 | 15873 |
| LSV5 | 3 | 110 | 435 | 0 | 734 | 90 |
| LSV5 | 3 | 391 | 64 | 0 | 141 | 614 |
| LSV5 | 0 | 0 | 0 | 0 | 2 | 1 |
| LSV5 | 11 | 281 | 699 | 200 | 438 | 11 |
| LSV5 | 0 | 0 | 1 | 0 | 0 | 3 |
| LSV5 | 184 | 0 | 2211 | 0 | 15702 | 230 |
| Sacbrood | 45 | 8 | 13 | 10 | 1885 | 32 |
| SBPV | 0 | 0 | 0 | 0 | 0 | 0 |
| Tobacco_ringspot_virus | 0 | 0 | 0 | 0 | 0 | 0 |
| Varroa_Macula_like | 0 | 0 | 0 | 0 | 0 | 3 |
| VDV | 1112041 | 1213796 | 550109 | 177304 | 11614 | 4703331 |
|  |  |  |  |  |  |  |
| **Symbionts** |  |  |  |  |  |  |
| Overall bacterial load | 600967 | 70382 | 20793 | 116926 | 14449 | 598188 |
| *Candidatus Schmidhempelia bomb*i | 8871 | 3716 | 2962 | 1706 | 265 | 5041 |
| Frischella | 37 | 10 | 4 | 5 | 2 | 20 |
| *Gilliamella apicola* | 45087 | 127562 | 147515 | 47120 | 71743 | 46403 |
| *Lactobacillus spp.* | 314 | 82 | 16 | 29 | 8 | 104 |
| *Snodgrasella alvi* | 41237 | 60276 | 49704 | 22323 | 58094 | 65751 |
|  |  |  |  |  |  |  |
| **Fungi** |  |  |  |  |  |  |
| *Ascosphaera apis* | 2031 | 728 | 400 | 618 | 58 | 862 |
| *Nosema ceranae* | 624056 | 437735 | 327516 | 1302005 | 597540 | 704281 |
|  |  |  |  |  |  |  |
| **Parasites** |  |  |  |  |  |  |
| *Acarapis spp.* (*Acari*) | 0 | 0 | 0 | 0 | 0 | 0 |
| *Tropilaelaps claerae* (*Acari*) | 0 | 0 | 0 | 2 | 0 | 1 |
| *Apocephalus* (*Phoridae*) | 0 | 0 | 0 | 0 | 0 | 0 |
|  |  |  |  |  |  |  |
